# Bridging worlds: connecting glycan representations with glycoinformatics via Universal Input and a canonicalized nomenclature

**DOI:** 10.1093/bioadv/vbaf310

**Published:** 2025-12-01

**Authors:** James Urban, Roman Joeres, Daniel Bojar

**Affiliations:** Department of Chemistry and Molecular Biology, University of Gothenburg, Gothenburg 40530, Sweden; Wallenberg Centre for Molecular and Translational Medicine, University of Gothenburg, Gothenburg 40530, Sweden; Department of Chemistry and Molecular Biology, University of Gothenburg, Gothenburg 40530, Sweden; Wallenberg Centre for Molecular and Translational Medicine, University of Gothenburg, Gothenburg 40530, Sweden; Saarbruecken Informatics Campus, Saarland University, Saarbruecken 66123, Germany; Helmholtz Institute for Pharmaceutical Research Saarland, Helmholtz Center for Infection Research, Saarbruecken 66123, Germany; Department of Chemistry and Molecular Biology, University of Gothenburg, Gothenburg 40530, Sweden; Wallenberg Centre for Molecular and Translational Medicine, University of Gothenburg, Gothenburg 40530, Sweden

## Abstract

**Motivation:**

As the field of glycobiology has developed, so too have different glycan nomenclature systems. While each system serves specific purposes, this multiplicity creates challenges for usability, data integration, and knowledge sharing across different databases and computational tools.

**Results:**

We present a practical framework for automated nomenclature conversion that takes any glycan nomenclature as input without requiring declaration of the specific language and outputs a canonicalized IUPAC-condensed format as a standardized representation. Our implementation handles all common nomenclatures including WURCS, GlycoCT, IUPAC-condensed/extended, GLYCAM, CSDB-linear, LinearCode, GlycoWorkbench, GlySeeker, Oxford, and KCF, along with common typos, and manages complex cases including structural ambiguities, modifications, uncertainty in linkage information, and different compositional representations. This Universal Input framework can translate more than 10 nomenclatures in <1 ms per glycan, tested on over 150 000 sequences with 98%–100% coverage, enabling seamless integration of existing glycan databases and tools while maintaining the specific advantages of each representation system.

**Availability and implementation:**

Universal Input is implemented within the glycowork Python package, available at https://github.com/BojarLab/glycowork and our web app https://canonicalize.streamlit.app/.

## 1 Introduction

Glycans, complex carbohydrates attached to proteins and lipids, play crucial roles in numerous biological processes, including cell signaling, immune response, and disease progression (Varki 2017). As glycobiology has evolved over the past decades, so too have nomenclature systems used to represent these intricate molecules (Aoki-Kinoshita 2019). This diversity of representational systems reflects the varied needs of different scientific communities working with glycans, from carbohydrate chemists requiring precise atomic configurations, over glycoinformaticians working with unique and computationally tractable formats, to systems biologists analyzing glycome changes at scale.

While each nomenclature serves specific purposes, the resulting fragmentation creates significant challenges for data integration, knowledge sharing, and interdisciplinary collaboration. Experimentalists often favor nomenclatures optimized for human readability and manual annotation, such as the Oxford notation for N-glycans (Rudd *et al.* 1997) or IUPAC-condensed formats (McNaught 1996) that are frequently used (Jin *et al.* 2023). In contrast, computational glycobiologists often require machine-readable formats such as WURCS (Matsubara *et al.* 2017) or GlycoCT (Herget *et al.* 2008) that capture structural details with formal precision. Other nomenclatures, used in various niches of the glycosciences, include LinearCode (Banin *et al.* 2002), CSDB Linear (Toukach and Egorova 2020), or GlycoWorkbench (Ceroni *et al.* 2008). This divergence has created a gulf between manual experimental work and computational glycoinformatics that impedes the field’s advancement, both for information exchange and for accumulating large datasets required for AI models (Bojar and Lisacek 2022).

While individual converters exist (Klein and Zaia 2019; Tsuchiya *et al.* 2019), databases utilizing different nomenclature systems can remain siloed, software tools become nomenclature-specific, and researchers must master multiple representational languages to access the full breadth of glycobiology knowledge. Inconsistencies, typos, and dialects across different research communities further compound this challenge. This leads to a common trend in the glycosciences, in which each cluster of research groups independently develops a suite of methods that are tailored to their nomenclature preferences and data format peculiarities.

The main reasons for this plethora are cognitive and practical advantages that different representations offer for specific use cases. IUPAC-condensed notation, e.g. offers an intuitive balance between human readability, easy editability, and structural precision that makes it particularly valuable for manual annotation. Meanwhile, formats such as WURCS excel at representing structural ambiguities but present a steeper learning curve for human interpretation.

We here propose to leverage these strengths through a Universal Input framework, capable of letting users work in any nomenclature they choose by automatically inferring any common nomenclature format, followed by its conversion into the widely used IUPAC-condensed format. Closely connected to this approach is the adoption of a canonicalized version of the IUPAC-condensed format, described here, as a standardized intermediate representation, exhibiting the best of both worlds. While standard IUPAC-condensed notation suffers from non-injectivity when mapping to other nomenclatures (i.e. several IUPAC-condensed strings can be mapped to the same WURCS glycan), and exhibits dialectical variations, these limitations can be easily and programmatically addressed through consistent canonicalization rules that we propose here, which take the burden of nomenclature conformity and validity from the user and place it on reactive and performant algorithms. We show further that this implementation is faster, more memory-efficient, and broader than current alternatives.

Our framework accommodates all common glycan nomenclatures and ID systems, including typical typographical variations and errors, while handling structural ambiguities, modifications, and uncertainty in linkage information. Additionally, it processes compositional representations that specify monosaccharide content without fully defined structures, which represents a common output from mass spectrometry-based glycomics (Ruhaak *et al.* 2018). Since most glycoinformatics resources support IUPAC-condensed, this means that users of all nomenclatures gain access to the resources across the entire field, as well as become capable of inspecting and combining datasets from other research groups, fostering both synergy and standardization across glycobiology.

By bridging the gap between manual experimental notation and computational glycoinformatics, this Universal Input framework enables researchers to work in their preferred nomenclature, while facilitating seamless data integration across the glycobiology ecosystem. Universal Input is implemented, and widely used, in the open-source Python library glycowork (Thomès *et al.* 2021), yet can be easily integrated into any Python-based application, such as our herein developed web application (https://canonicalize.streamlit.app/). The resulting interoperability gained from Universal Input promises to accelerate discovery by connecting previously isolated data repositories, enabling cross-platform tool development, and fostering interdisciplinary collaboration across the diverse communities studying glycan structure and function.

## 2 Methods

### 2.1 Universal Input

Within the Universal Input system, a combination of dedicated parsers and a common stem comprise the *canonicalize_iupac* function. We decompose this into several blocks, further described below: (i) nomenclature detection via hooks, (ii) individual parsers into basic IUPAC-condensed, (iii) a common stem to streamline IUPAC-condensed variations and general clean-up, and (iv) a branch canonicalization algorithm to yield canonicalized IUPAC-condensed sequences. The Universal Input system, as described here, is available in glycowork (v1.7.0+), the glycoworkGUI (v1.7.0+), and a dedicated web interface (https://canonicalize.streamlit.app/). If used via Python, Python ≥3.10 is required.

### 2.2 Nomenclature detection

First, common names (e.g. “LacNAc”) are accessed in a glycowork-stored dictionary with a runtime complexity of O(1), for which we ignore capitalization and spaces. This also means that expanding this dictionary with new synonyms will never worsen performance and makes our pipeline robust to future additions. Next, the presence of specific hooks in the glycan sequence (see Table 1, available as supplementary data at *Bioinformatics Advances* online) will trigger the *linearcode_to_iupac*, *glyseeker_to_iupac*, *iupac_extended_to_condensed*, *glycoct_to_iupac*, *wurcs_to_iupac*, *glycam_to_iupac*, *GAG_disaccharide_to_iupac*, *glytoucan_to_glycan*, *nglycan_stub_to_iupac*, *kcf_to_iupac*, *glycoctxml_to_iupac*, or *oxford_to_iupac* parser in *glycowork.motif.processing*. The usage of yet-unsupported nomenclatures, such as SMILES, is detected via the *check_nomenclature* function and will lead to an appropriate error.

### 2.3 Individual parsers

All parsers heavily use regular expressions and other code operations to convert the original nomenclature into a flat string resembling IUPAC-condensed nomenclature. WURCS, GlycoCT, GlycoCT XML, KCF, and, to some extent, Oxford are first parsed into dictionaries of connections and building blocks, which are then used to construct the final sequence. WURCS parsing in particular relies on a mapping of valid tokens, stored within glycowork. We stress that the individual parsers are not meant to be used in isolation because, by design, they do not result in clean and final IUPAC-condensed nomenclature. For this, we use the common stem of *canonicalize_iupac*, described below, to perform any shared operations (such as canonicalizing the usage of parentheses and brackets etc.).

### 2.4 Cleaning IUPAC-condensed sequences in a common stem

In general, the common stem of *canonicalize_iupac* uses extensive regular expressions to capture, homogenize, and standardize nomenclature syntax. As a first step, common token variants are detected and replaced (e.g. NeuAc → Neu5Ac or β → b). Other operations here include sanitizing variations in the placement of anomeric and enantiomeric indicators, in the usage of dashes (or not) in linkages, in the convention of explicitly declaring the starting carbon or not, and many others. Another common theme here is the standardization of linkage ambiguity (e.g. occasionally noted as: Mana-, Man-, Mana1-, Man[, Man-[, ManMan, and many other variations). Next, we standardize the usage of parentheses vs. brackets. If parentheses are used for branches, this is changed to the conventional style of parenthesis = linkage, bracket = branch. Similarly, we standardize the usage of glycan modifications, where we encountered (and fixed) variants such as: [4S]Gal, Gal? S, [S]Gal, SGal, S-Gal, and many more. Next, variants of denoting uncertain substituents (i.e. “we know there is a sialic acid in this glycan but not where”) are standardized to the format of curly bracket = floating substituent. Thereafter, the common stem automatically checks for chemical impossibilities (e.g. linkages where no acceptor hydroxyl group is available or multiple linkages ending in the same acceptor hydroxyl group) and sets the corresponding linkages to? -containing wildcards that resolve the impossibility. We note that this common stem also converts CSDB-linear into IUPAC-condensed, both because we did not find a unique hook for a CSDB-specific parser but also because many of the operations are shared between the two nomenclatures.

### 2.5 Canonicalization of branch ordering

If the used glycan sequence at the end of the common stem contained branches, we used a branch canonicalization algorithm that resulted in a unique IUPAC-condensed sequence per glycan. This is done by converting the glycan into a NetworkX-based directed graph (via *glycowork.motif.graph.glycan_to_nxGraph*), reordering branches on the graph level, and then translating that graph back into a canonicalized IUPAC-condensed sequence via the *glycowork.motif.graph.graph_to_string* function (using the *canonicalize_glycan_graph* function). For the actual branch canonicalization, we use a recursive depth-first traversal approach. First, it calculates values using post-order traversal (where children are processed before parents), and then builds the canonical graph using pre-order traversal (where parents are processed before children). The sorting of branches happens at each node during this depth-first process, prioritizing the longest paths (i.e. the branch with the longest chain of monosaccharides, recursively). When two branches have equal path lengths, tie-breaking is performed first by the linkage number of the branch parent (lower numbers are preferred; e.g. Man(a1-3) branch before Man(a1-6) branch, in *N*-glycans), with integer linkages prioritized over wildcards. If linkage numbers are also equal, branches are further distinguished alphabetically by comparing the minimum leaf labels (or proceeding down the branch until an alphabetic difference is detected), ensuring a completely deterministic ordering.

### 2.6 Collecting glycan sequences for benchmarks

For WURCS sequences, we gathered all sequences labeled “Linkage-defined saccharide” that were available on GlyCosmos (Yamada *et al.* 2020) (April 2025). For GlycoWorkbench, we gathered all available sequences from https://gitlab.com/glycoinfo/glycoworkbench. For Oxford, we gathered representative examples from the following GlycoPOST IDs (Watanabe *et al.* 2021): GPST000308, GPST000318, GPST000421, GPST000473, GPST000494. For GLYCAM and GlycoCT, we gathered all sequences that were available on GlycoShape (Ives *et al.* 2024) (May 2025). For GlySeeker (Xiao *et al.* 2018), we gathered sequences from various GlycoPOST IDs. For CSDB-Linear, we were supplied with a list of representative sequences from Dr Tom Stanton (https://github.com/BojarLab/glycowork/issues/76). For IUPAC-condensed, we gathered all sequences in the *df_glycan* dataset in glycowork (v1.7.0) (Thomès *et al.* 2021). For KCF, we randomly gathered 1000 sequences from KEGG.

## 3 Results

### 3.1 Canonicalized IUPAC-condensed to balance computation and readability

**Figure 1.**
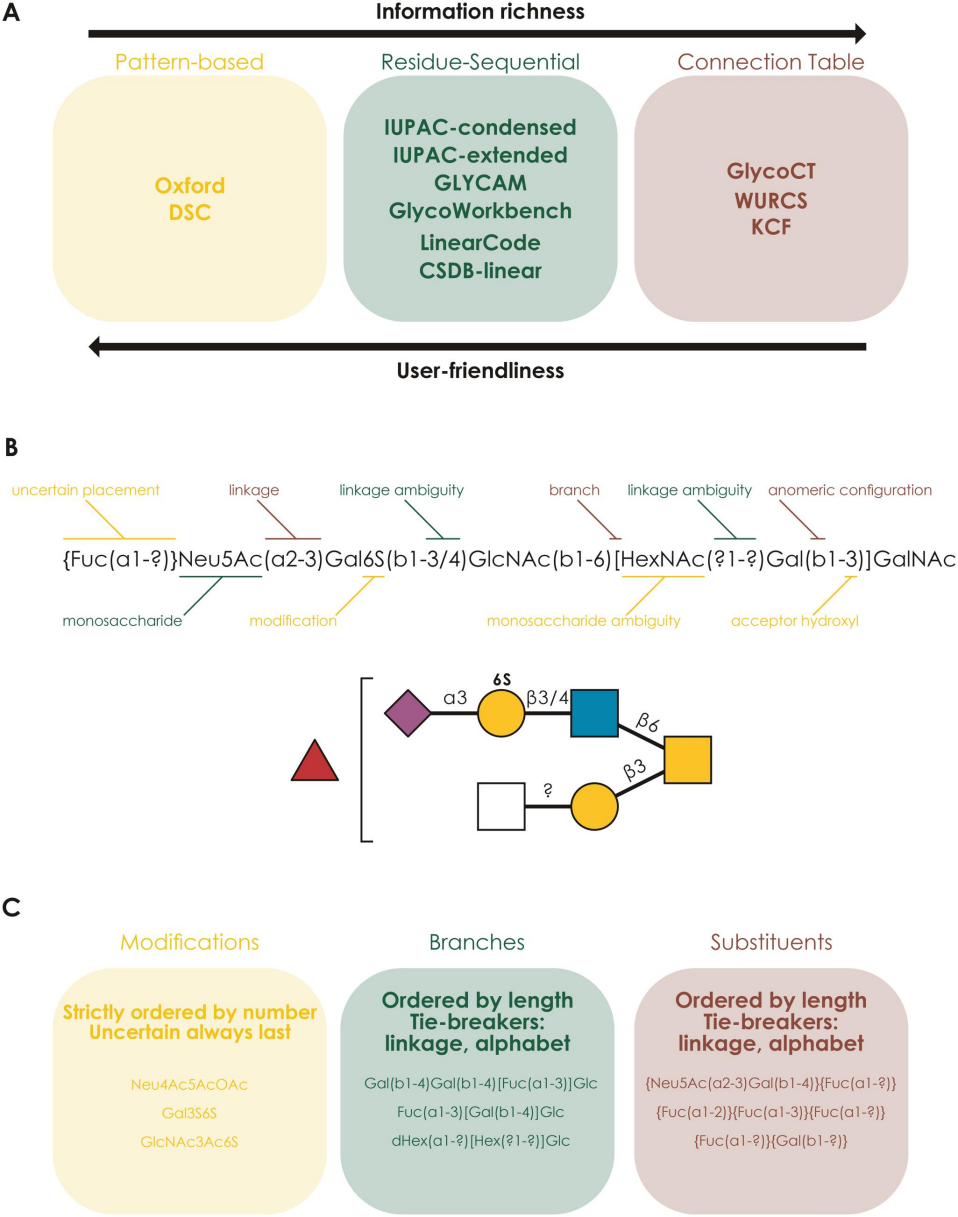
IUPAC-condensed as a balanced nomenclature for glycobiology. (A) An overview of common glycan nomenclatures and their properties. (B) Anatomy of the IUPAC-condensed nomenclature, with the example of an *O*-glycan, visualized in the Symbol Nomenclature For Glycans (SNFG) via GlycoDraw (Lundstrøm *et al.* 2023b). (C) Ordering guidelines for converting a regular IUPAC-condensed glycan into canonicalized IUPAC-condensed, as defined here. We emphasize that we do not intend this as a user manual but rather a transparent explanation of the ordering guidelines employed by our canonicalization algorithms.

While many glycan nomenclatures exist, we set out with the hypothesis that residue-focused nomenclatures offer the best balance between human-readability and computational processing (Fig. 1A). As outlined above, IUPAC-condensed has been developed as a human-readable notation that describes glycans while still being easily processable by computers (Fig. 1B), as we have shown repeatedly (Lundstrøm *et al.* 2023a, Thomès *et al.* 2023, Bennett and Bojar 2024, Bennett *et al.* 2025). The latter is also already amply demonstrated by the glycowork-internal parser (Thomès *et al.* 2021) and the grammar laying the foundation of the IUPAC-to-SMILES translator GlyLES (Joeres *et al.* 2023). Briefly, IUPAC-condensed uses common shorthand monosaccharide names, where only the uncommon form is further specified (e.g. Glc instead of D-Glcp), which are connected via parenthesis-enclosed linkages containing (i) the anomeric configuration, (ii) the anomeric carbon, and (iii) the recipient hydroxyl group on the subsequent monosaccharide, such as in Gal(b1-4)Glc. Branches are enclosed in square brackets, which can be nested, while residues with uncertain connection to the remaining glycan are enclosed in curly brackets. Modifications are typically listed as a number (indicating the carbon at which this modification is found), followed by a shorthand notation of the modification (e.g. Gal6S). Ambiguities can be further expressed on the monosaccharide level (e.g. Hex instead of Gal) and on the linkage level (e.g. a1-?,? 1-4, or the more granular a1-3/4).

One key property of the classic IUPAC-condensed notation is that multiple semantically valid notations exist for a single glycan, e.g. Gal(b1-3)GalNAc(b1-4)[Neu5Ac(a2-3)]Gal(b1-4)Glc and Neu5Ac(a2-3)[Gal(b1-3)GalNAc(b1-4)]Gal(b1-4)Glc. There are also countless dialects and personal notational preferences (a flexibility that has led to the nomenclature’s popularity among experimentalists) that need to be considered. To simplify working with IUPAC-condensed computationally, we thus set out to canonicalize its notation, defining one unique description per glycan. We emphasize here that these canonicalizations are not meant as a prescriptive burden to users of IUPAC-condensed. Rather, they are intended as algorithmically enforceable routines, to map any arbitrary IUPAC-condensed starting point into a unique, canonicalized string (that is still human-readable and editable). The canonicalization of IUPAC-condensed consists of two main parts: (i) enforcing “standard” IUPAC-condensed (i.e. remedying dialects and typos) and (ii) ordering modifications, floating substituents, and branches in a deterministic manner (Fig. 1C).

While Gal(b1-4)Glc, Galb1-4Glc, and Galb4Glc all describe lactose, only the first one would be considered standard IUPAC-condensed. Within glycowork, and in the *canonicalize_iupac* function described below, we employ extensive regular expression operations to guarantee the proper namespace of monosaccharides, modifications, linkages, as well as many other variations. While we discuss other nomenclatures below, this already allowed us to homogenize the great diversity within the realm of IUPAC-condensed, converting them into standard IUPAC-condensed nomenclature, including important dialects such as CarbBank (Doubet and Albersheim 1992).

Next to following these conventional IUPAC-condensed guidelines (McNaught 1996), our canonicalization algorithms (see Section 2) also follow strict ordering guidelines that circumvent the inherent ambiguity of the nomenclature. We here emphasize that the particular choice of ordering is almost irrelevant, and the main benefit derives from having guidelines that can be (algorithmically) enforced from any starting point. With the example of branch ordering (Fig. 1C), we employ a recursive, graph-based, approach that iteratively designates the longest (i.e. most monosaccharides) branch as the main chain, with several tiebreakers (e.g. linkage at the branchpoint) that ensure a unique ordering, which, on average, minimizes nested branches.

We note that the convenience of having a guaranteeable and regular nomenclature format has allowed us to implement the extremely efficient glycan processing operations within glycowork, such as crafting a glycan-specific regular expression system (Bennett and Bojar 2024), and we are convinced this set-up will spur more advances in the glycosciences.

### 3.2 The universal Input system infers and auto-converts all common nomenclatures

**Figure 2.**
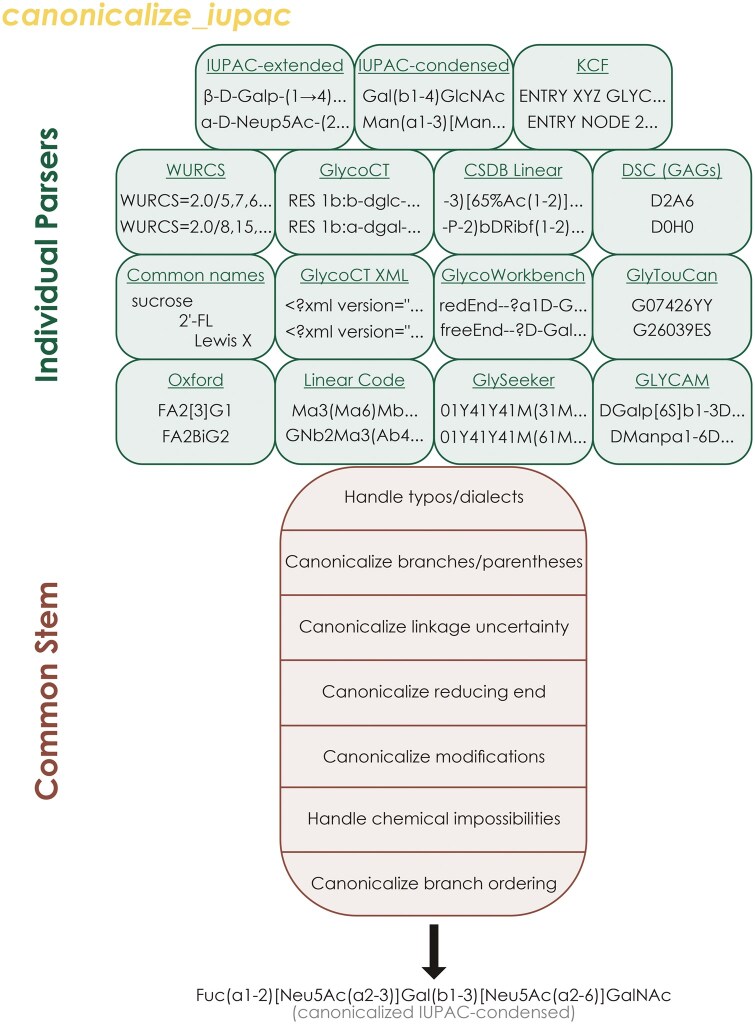
Universal Input is a potent platform to enable users to work in any common glycan nomenclature. Glycan sequences used as input for *glycowork.motif.processing.canonicalize_iupac* first are searched for unique hooks indicating a certain nomenclature, after which they are dispatched to the dedicated parser for that nomenclature, followed by all sequences passing through the common stem, in which dialectical variation in syntax, monosaccharide name space, modification handling, and branch ordering are sanitized and canonicalized to arrive at a unique canonicalized IUPAC-condensed string per molecule.

The main issue with proposing new nomenclature variants inevitably is user conformity (and, hence, adherence to the canonicalized nomenclature). We propose to solve this issue here with our main advance of this work, the Universal Input system (Fig. 2), in which any deviation from this canonicalized nomenclature (e.g. different nomenclatures, nomenclature dialects, arbitrary branch ordering, typos) is automatically fixed, such that users do not need to comply with (or even be aware of) syntactic rules, as they can be algorithmically enforced from any common starting point, without appreciable computational overhead as we show below.

In general, such a system requires two operations: (i) a robust means to detect a deviation and (ii) an algorithmic solution to remedy said deviation. We distinguish here between different nomenclatures and notational variance of IUPAC-derived and similar nomenclatures. For dedicated nomenclatures (Fig. 2), we identified unique string- or regular expression-patterns (“hooks”; see Table 1, available as supplementary data at *Bioinformatics Advances* online) that allowed us to call a specific parser for that nomenclature, which we newly implemented for this work. Generally, the consolidation of “post-processing” operations into a common stem has allowed us to keep parsers relatively lightweight and fast to construct, as their task was merely to convert sequences into anything resembling IUPAC nomenclature, without strict requirements on particular notation, making this system easily extendable to new nomenclatures.

**Figure 3.**
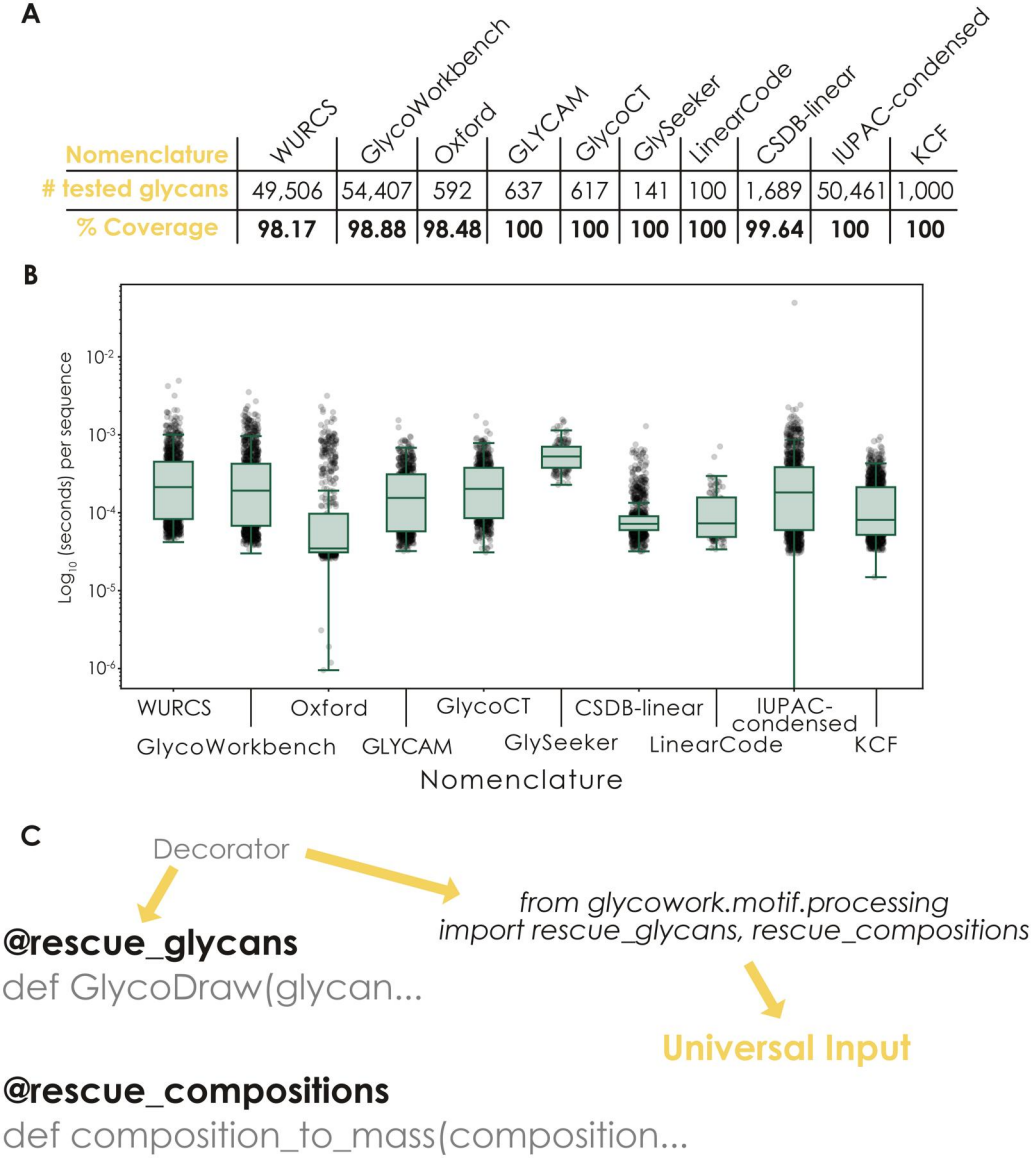
Universal Input is robust, performant, and readily usable anywhere in Python. (A) Coverage of common nomenclatures by Universal Input. For the glycan nomenclatures of WURCS, GlycoWorkbench, Oxford, GLYCAM, GlycoCT, GlySeeker, LinearCode, CSDB-linear, IUPAC-condensed, and KCF, we have assembled a set of representative sequences (Table 2, available as supplementary data at *Bioinformatics Advances* online) and tested them with the *canonicalize_iupac* function, shown here as the success rate of this function. (B) Speed test of Universal Input. For the sequences mentioned in (A), we then also timed the execution speed of the *canonicalize_iupac* function per nomenclature, visualized as box plots (the line being the median values, with box edges indicating quartiles, and whiskers indicating the remaining data distribution up to the 95% confidence interval), overlaid with the results of up to 1000 randomly chosen sequences as a swarm plot. We report average runtimes of 0.2 ms per glycan, when run on a MacBook M3 Pro (1 ms per glycan, when run on a default Google Colaboratory instance with a Intel Xeon CPU @ 2.20 GHz). (C) Enabling Universal Input in any Python code. Using the Universal Input decorators for sequences (*rescue_glycans*) and compositions (*rescue_compositions*), functions in Python can be equipped with Universal Input capabilities with a single line of code, imported from glycowork.

We note that, tested on >150 000 sequences (Table 2, available as supplementary data at *Bioinformatics Advances* online; see also Fig. 3 below), our hooks for nomenclature detection are empirically robust to typically used sequences and all common nomenclatures. Universal Input also supports all common glycan ID systems (GlyTouCan, GlyConnect). The common stem of *canonicalize_iupac*, after the parsers, then mainly used carefully crafted regular expressions for many common deviations (e.g. in monosaccharide namespace, linkage notation, modification usage, etc.), collected and refined over the past 3 years. Lastly, branching order was algorithmically canonicalized, based on the rules mentioned above, resulting in a unique string per molecule that is (i) human-readable, (ii) easily editable, and (iii) amenable to be analyzed by performant analyses such as presented within glycowork. This entire workflow is consolidated in the single *canonicalize_iupac* function within glycowork (as well as in easily exportable decorators, as mentioned below). Even more importantly, this function (and its connected decorator) is widely used in glycowork itself, making the package compatible with all common nomenclatures, and, e.g. allows users to draw SNFG-depictions of glycans by calling the *GlycoDraw* function on WURCS sequences. We emphasize that *canonicalize_iupac* (and thus Universal Input) does not require any other information than a string-based glycan sequence, as everything else will be automatically inferred, making it very user-friendly and robust.

Further, while the line between sequences and compositions can be blurry and *canonicalize_iupac* supports some structured compositions such as “(Hex)3 (HexNAc)1 (NeuAc)1 + (Man)3(GlcNAc)2,” we also developed the *canonicalize_composition* function to support the great diversity of composition formats used in the academic literature (e.g. H5N4F1A2 or Hex5HexNAc4Fuc1Neu5Ac2; Fig. 1, available as supplementary data at *Bioinformatics Advances* online), which we also use within glycowork to support mass calculations and other operations with any common composition nomenclature.

We next set out to establish the coverage and robustness of our Universal Input system by testing the *canonicalize_iupac* function on up to 50 000 sequences per nomenclature (Table 2, available as supplementary data at *Bioinformatics Advances* online), resulting in coverage values ranging from 98% to 100% (Fig. 3A), with representative error cases shown in Table 3, available as supplementary data at *Bioinformatics Advances* online. The most common error cases represented rare unsupported tokens (WURCS, Oxford), SMILES substituents (CSDB-linear), or alternative ways to denote narrow ambiguities (GlycoWorkbench). We confirm that unsupported tokens are exclusively rare and do not occur in more than five known glycans. We note that for nomenclatures such as Oxford, without a dedicated database, we had to resort to assembling our own benchmark dataset from individual publications. Coverage, error sources, and timing (see below) can be reproduced and tested with a Google Colaboratory notebook that can be found at https://colab.research.google.com/drive/1kq_FDZdWTrVp64ztvPXIBCD0r-8lsMsF? usp=drive_link.

Since the Universal Input functions often run in the background for operations within glycowork, we needed to ensure that they still stayed as performant as possible for a seamless user experience. We thus benchmarked the speed of *canonicalize_iupac* on the sequences used for our coverage test above and, despite numerous regular expression operations and other procedures, can report an average processing time of <0.2 ms per glycan on regular consumer hardware (Fig. 3B), regardless of nomenclature. We also compared *canonicalize_iupac* to the format conversion within glypy (Klein and Zaia 2019) on WURCS, GlycoCT, and LinearCode (since other nomenclatures were not supported on glypy) as well as a local install of GlycanFormatConverter (Tsuchiya *et al.* 2019) on those nomenclatures plus IUPAC-condensed. On average, Universal Input was more performant than both GlycanFormatConverter and glypy (Fig. 2A, available as supplementary data at *Bioinformatics Advances* online). Further, our Universal Input platform was strictly faster for converting sequences than GlycanFormatConverter, faster or equivalent to glypy (depending on the nomenclature; Fig. 2B, available as supplementary data at *Bioinformatics Advances* online), and offered more features than both alternatives (Fig. 2C, available as supplementary data at *Bioinformatics Advances* online). Since both GlyCosmos and GlyGen internally rely on GlycanFormatConverter, we thus conclude that we have compared our work with all currently available and relevant multi-nomenclature conversion platforms.

Lastly, while we both offer Universal Input to users and elevate glycowork’s functionality with it, we reasoned that it might be modular enough to be integrated into any Python package related to glycobiology. For this, we offer two options: (i) importing the actual *canonicalize_iupac*/*canonicalize_composition* functions, for granular functional control over where exactly canonicalization should occur, or (ii) the new *rescue_glycans*/*rescue_compositions* decorators. These decorators can be prepended before every function that uses glycan strings (or lists of strings) as input and automatically equip that function with Universal Input capabilities (Fig. 3C).

We have tested this system recently in our CandyCrunch (Urban *et al.* 2024) and GlyContact (Thomès *et al.* 2025) packages and achieved Universal Input for each package with just a few lines of code, with full control over which functions should and should not benefit from this system. In the case of CandyCrunch, this has allowed us to rapidly gather disparate data from sources such as GlycoPOST (Watanabe *et al.* 2021), whereas in GlyContact, Universal Input facilitates the exploration of glycan 3D structures by using any nomenclature of choice, including common names such as “LacNAc” etc., which is especially important as structural glycobiologists often use the, otherwise poorly supported, GLYCAM nomenclature. We thus conclude this section by arguing that Universal Input makes the glycowork package an even more attractive starting point for glycoinformatics, both in itself, as well as in building capacity for constructing downstream Python applications.

### 3.3 Making Universal Input accessible yields new opportunities for glycobiology

**Figure 4.**
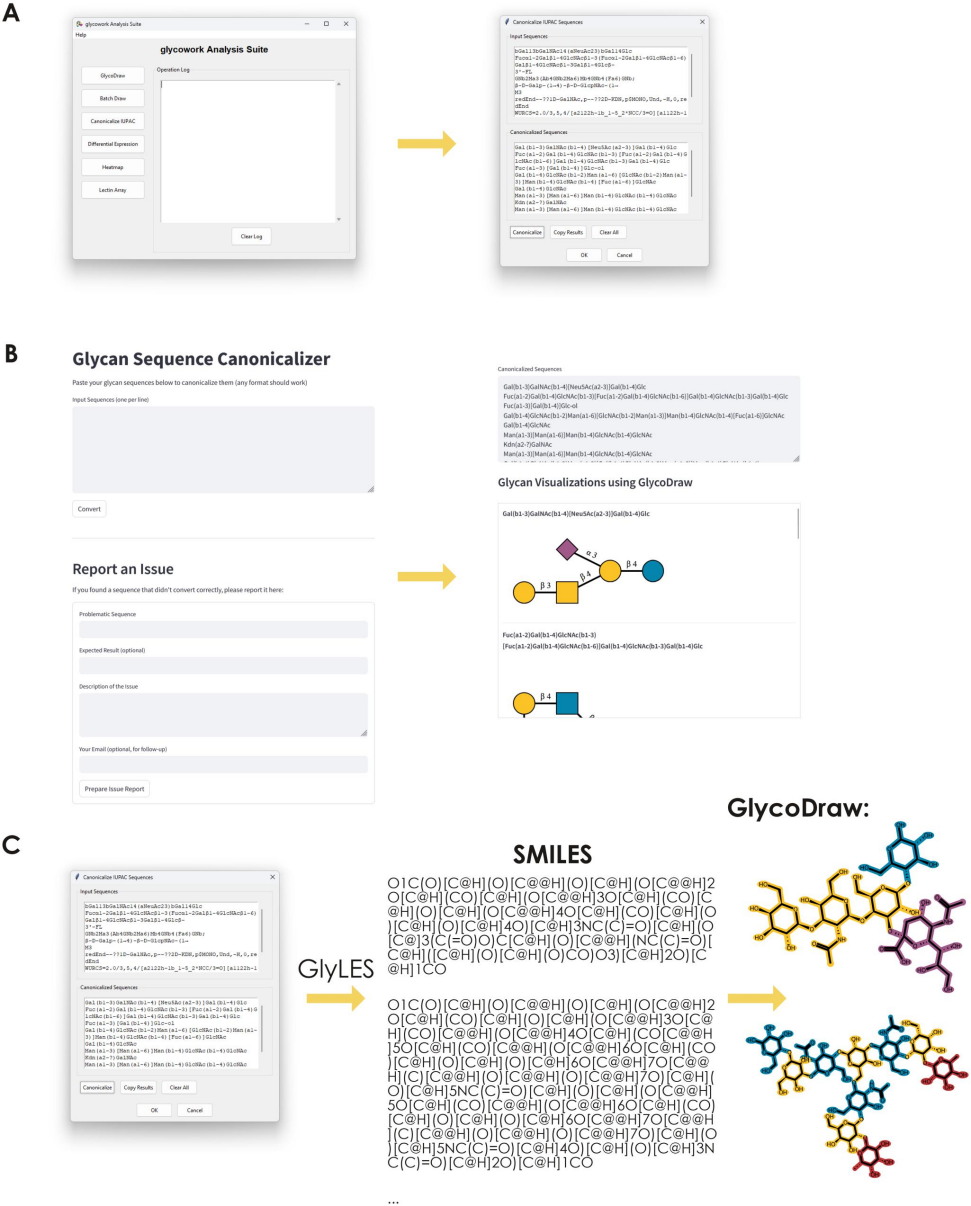
Universal Input is easily accessible and unlocks access to chemoinformatics. (A) Using Universal Input via the glycoworkGUI (v1.7.0). The graphical user interface of glycowork exposes the *canonicalize_iupac* function as “Canonicalize IUPAC,” which allows users to input a number of sequences in any nomenclature format and copy the output anywhere they choose. (B) Using Universal Input via our web application. On https://canonicalize.streamlit.app, users can input any number of glycan sequences in any format, convert them into canonicalized IUPAC-condensed with a single click, and copy them or view them via GlycoDraw-drawn SNFG depictions in a scroll box. (C) Using Universal Input to convert any glycan nomenclature into SMILES strings. Any canonicalized IUPAC-condensed sequence obtained from our Universal Input platform can be converted into SMILES strings using the *glycowork.motif.processing.IUPAC_to_SMILES* function (which relies on the GlyLES converter we previously developed). This then interfaces with existing chemoinformatic functionality, such as drawing glycans on an atomic level using GlycoDraw within glycowork.

Since *canonicalize_iupac* operates on a per-glycan level, there is no need for users to exclusively use one nomenclature. Datasets with any mixture of nomenclatures can be readily used for any workflow within glycowork (Fig. 3, available as supplementary data at *Bioinformatics Advances* online). We can envision at least two use-cases for which this is useful: (i) any comparative analysis endeavor, up to a full meta-analysis, will have gathered data in all kinds of nomenclatures, which is easily handled with this functionality, and (ii) datasets containing one or a few glycans with not (yet) supported sequence features, such as unusual modifications, can still largely be analyzed with most of the functionality within glycowork. To illustrate this, we have used our Universal Input pipeline to analyze glycosphingolipid (GSL) data from several publications (Huang *et al.* 2022, Lee *et al.* 2024, Moh *et al.* 2024, Schindler *et al.* 2025) in various nomenclatures (e.g. GSL common names and GlycoWorkbench), which has allowed us to ascertain similarities and differences between human and mouse brain GSL levels (Fig. 4, available as supplementary data at *Bioinformatics Advances* online).

We realized that this platform of not only converting nomenclatures but also canonicalizing conventional nomenclatures, as well as cleaning typos and other errors, could be generally useful for the broader audience of non-python glycobiologists. Therefore, we exposed the *canonicalize_iupac* functionality via our no-code graphical user interface (GUI) for glycowork that is freely available for Windows and Mac via https://github.com/BojarLab/glycowork/releases (Fig. 4A).

To expand the reach of our Universal Input platform even further, we then also developed a web application using Streamlit that allowed users to rapidly copy/paste their to-be-converted sequences in plain text (https://canonicalize.streamlit.app/; Fig. 4B). For maximum utility, this web-app also used our GlycoDraw (Lundstrøm *et al.* 2023b) platform to visualize converted sequences in a SNFG format, allowing users to quickly assess whether the conversion worked as expected (or export SNFG drawings), even if they are unfamiliar with the canonicalized IUPAC-condensed nomenclature. We also added an error report form to this application, facilitating interaction with the community and further improvement of the Universal Input platform.

Lastly, we wanted to stress the lingua franca aspects of our canonicalized IUPAC-condensed approach by showcasing the connection to state-of-the-art glycoinformatics capabilities this facilitates. One example of this can be shown via integration with the GlyLES platform (Joeres *et al.* 2023), which converts IUPAC-condensed glycans into SMILES strings. These well-established chemical descriptions of molecules are not only opening doors to the entirety of chemoinformatics (RDKit etc.) but also directly enable mass calculations and other essential operations to characterize glycans. We stress that this is especially relevant to glycochemists, who are so far underserved by the biology-oriented nomenclatures in the glycosciences. *De facto*, our Universal Input platform makes every glycan nomenclature easily convertible into SMILES strings (without the need for dedicated GlycoCT-to-SMILES parsers etc.), by first converting it into canonicalized IUPAC-condensed as a ‘pivot language’ that is then compatible with approaches such as GlyLES (Fig. 4C). To demonstrate this, we have calculated and compared the normalized topological polar surface area (TPSA) for glycans in three databases (glycowork, GlyCosmos, GlycoWorkbench), written in three different nomenclatures (IUPAC-condensed, WURCS, GlycoWorkbench), demonstrating the potential of this approach (Fig. 5, available as supplementary data at *Bioinformatics Advances* online). All scripts and converted SMILES strings for this analysis can also be found here: https://drive.google.com/drive/folders/1xsTQvEw7YFPhst8XglccKbf9wDqsiV5f? usp=drive_link.

## 4 Discussion

The Universal Input framework presented here addresses several critical challenges in glycoinformatics while offering distinct advantages over existing approaches. While, in some cases, individual converters exist for glycan nomenclatures, no current approach handles as many nomenclatures simultaneously as Universal Input. This also includes converters for nomenclatures which do not have any conversion resource at the moment, such as Oxford. The offline functionality of our framework enables nomenclature conversion without requiring internet connectivity or external service dependencies. This feature ensures accessibility and delivers superior performance, with faster conversion speeds than both local and web-based alternatives.

Our approach also lays the groundwork for improved biocuration practices in glycobiology (Martinez *et al.* 2024). By providing a standardized method for nomenclature conversion, this framework enables more consistent annotation of glycan structures across databases and literature. Automated data mining approaches (Kahsay *et al.* 2025) could use Universal Input to accommodate common variations in glycan notations during data extraction. This standardization facilitates developing quality control mechanisms for detecting and correcting inconsistencies in glycan structure databases. Overall, we emphasize that our main goal is not to derive a theoretically perfect nomenclature, but to craft a system that is maximally useful in real-world practice, by taking the burden of nomenclature validity from the user. We also note that these biocuration capabilities of Universal Input have allowed us to accumulate large datasets to train and improve AI models such as CandyCrunch (Urban *et al.* 2024) (predicting glycans from mass spectrometry data) and LectinOracle (Lundstrøm *et al.* 2022) (predicting lectin-glycan interactions).

The modular architecture of our framework (rudimentary parsers + a common stem) represents another key advantage. Adding support for a new nomenclature system requires only the development of a rudimentary parser that converts to anything remotely close to IUPAC-condensed notation—often only a few lines of code—along with a “unique hook” detection mechanism that identifies the nomenclature from input strings. If no sufficiently performant hook can be identified, this is usually an indicator of strong IUPAC-condensed resemblance (e.g. CSDB-Linear) and can be handled by the common stem. This extensibility ensures that the framework can evolve alongside the field, accommodating both established and emerging nomenclature systems. Our modular design also allows for targeted improvements to specific parsers without disrupting the overall conversion pipeline, facilitating continuous refinement made possible by our open-source software.

We also present the canonicalized IUPAC-condensed format here, which is used as the front-end nomenclature in glycowork and the output of Universal Input. While the original IUPAC-condensed notation suffers from ambiguities and dialectical variations, our herein presented canonicalization rules establish a consistent interpretation that preserves intuitive structure, while enabling reliable computational processing. This standardized representation provides a foundation for powerful back-end applications, such as the glycan graphs used in glycowork (Thomès *et al.* 2021).

Despite these advances, several challenges remain. The complex and often inconsistent nature of glycan nomenclature systems means that edge cases will inevitably arise that require refinement of our conversion algorithms, which is why the open-source nature of our code is so crucial. The open-source model enables specialists across the entire field to contribute improvements, extending the coverage and accuracy of this framework. Community-driven development also helps distribute the maintenance burden, increasing the likelihood that Universal Input will remain viable as glycoinformatics continues to evolve.

Additionally, while our framework handles the syntactic aspects of nomenclature conversion effectively, addressing the semantic layer (i.e. ensuring that converted structures accurately represent the intended molecular reality) requires both active curation and ongoing validation against experimental data. Future work should focus on expanding our coverage of rare monosaccharides and unusual modifications in various nomenclatures while developing more sophisticated validation mechanisms to assess conversion accuracy or capture unphysiological structures.

## Supplementary Material

vbaf310_Supplementary_Data

## Data Availability

The data underlying this article are available in our Supplementary Tables. The Universal Input framework is available, actively used by, and accessible from glycowork (https://github.com/BojarLab/glycowork), v1.7.0+.
