# Supplementary material for: Bridging worlds: connecting glycan representations with glycoinformatics via Universal Input and a canonicalized nomenclature
Source: Bioinform Adv. 2025 Dec 1;5(1):vbaf310. doi: 10.1093/bioadv/vbaf310 (PMC12702141; doi:10.1093/bioadv/vbaf310)
Supplement: vbaf310_Supplementary_Data [file vbaf310_supplementary_data.zip › universal_input_SI.docx]

**Bridging Worlds: Connecting Glycan Representations with Glycoinformatics via Universal Input and a Canonicalized Nomenclature**

James Urban^1,2^, Roman Joeres^1,2,3,4^, Daniel Bojar^1,2,*^

^1^Department of Chemistry and Molecular Biology; University of Gothenburg; Gothenburg, 405 30; Sweden

^2^Wallenberg Centre for Molecular and Translational Medicine; University of Gothenburg; Gothenburg, 405 30; Sweden

^3^Saarbruecken Informatics Campus, Saarland University, Saarbruecken, 66123; Germany

^4^Helmholtz Institute for Pharmaceutical Research Saarland, Helmholtz Center for Infection Research, Saarbruecken, 66123; Germany

^*^Corresponding author

**Supplementary Figures**

**
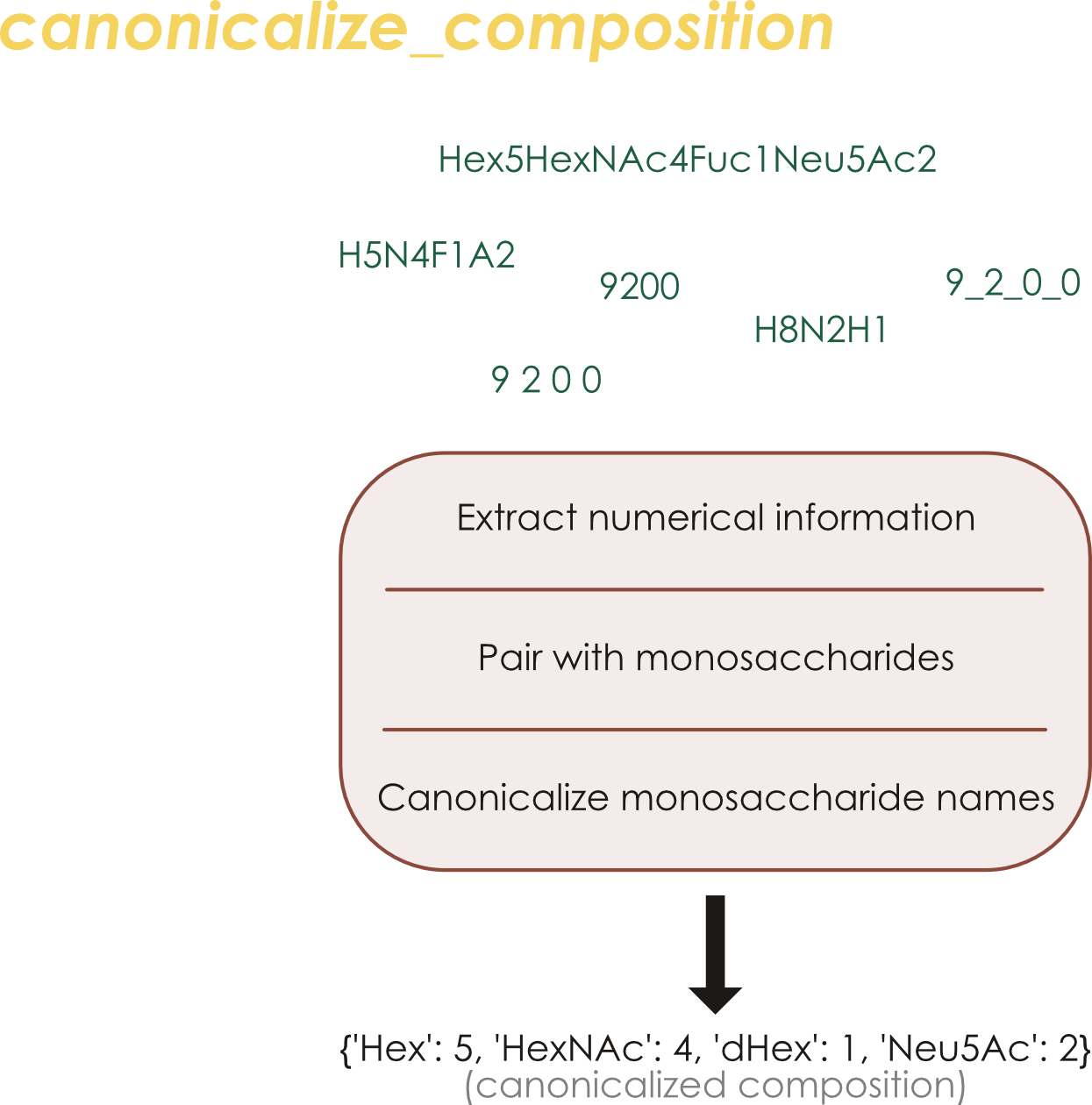
Supplementary Figure 1.** **Universal Input supports various composition notations.** Examples of supported composition formats are shown, together with the processing steps performed in *glycowork.motif.processing.canonicalize_composition*, leading to a standardized dictionary of type monosaccharide : quantity. We note that the monosaccharide namespace of the output is controlled and dictionaries are insertion-ordered to always result in the exact same dictionary for two glycans with identical compositions, allowing us to hash it for fast comparisons.


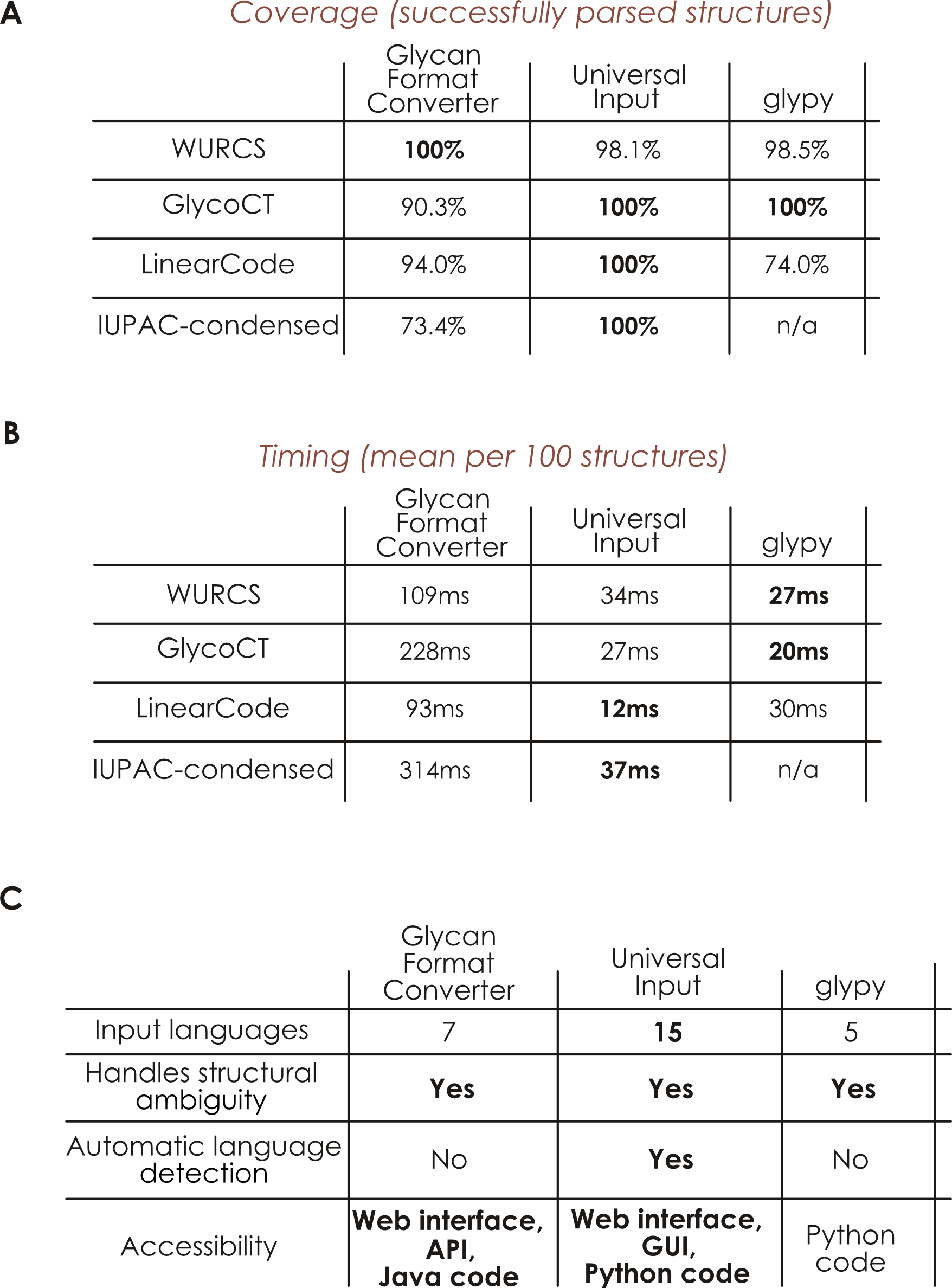


**Supplementary Figure 2.** **Universal Input is faster and more efficient than alternatives. A-B)** Comparing our benchmark sequences for WURCS, GlycoCT, LinearCode®, and IUPAC-condensed with the *glypy.io* module (v1.0.17), the *canonicalize_iupac* from glycowork (v1.7.0), and a local installation of GlycanFormatConverter (java) regarding coverage (A) and timing (B) on a MacBook M3 Pro. **C)** Feature comparison of the nomenclature conversion systems relevant for usage in Python. Best option for each row is bolded.


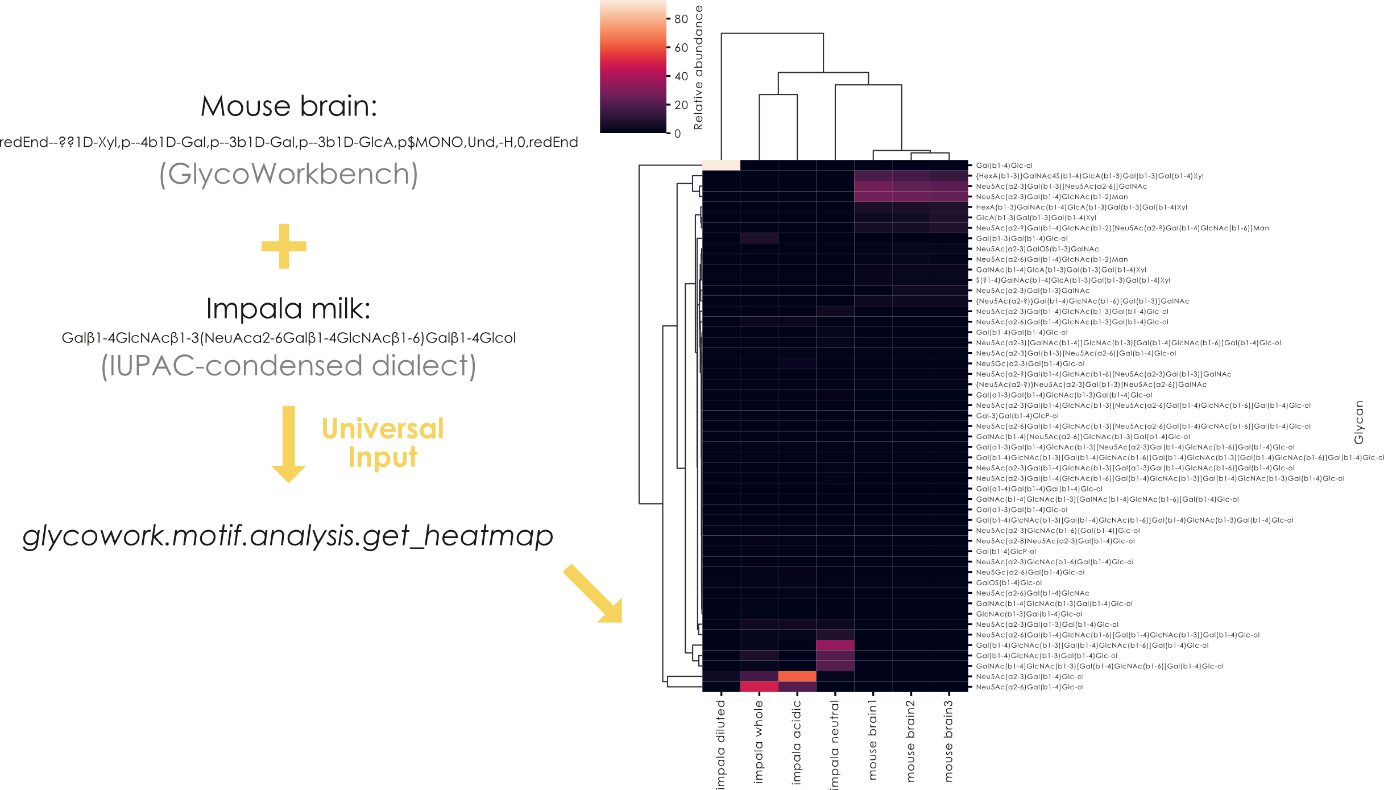


**Supplementary Figure 3.** **Universal Input supports mixing of nomenclatures.** Using mouse brain *O*-glycans in GlycoWorkbench (GPST000374) and impala free milk oligosaccharides in a dialect of IUPAC-condensed (GPST000317), we present a proof-of-concept experiment of using separate nomenclatures in the same dataset, used for hierarchical clustering via *glycowork.motif.analysis.get_heatmap*.

**
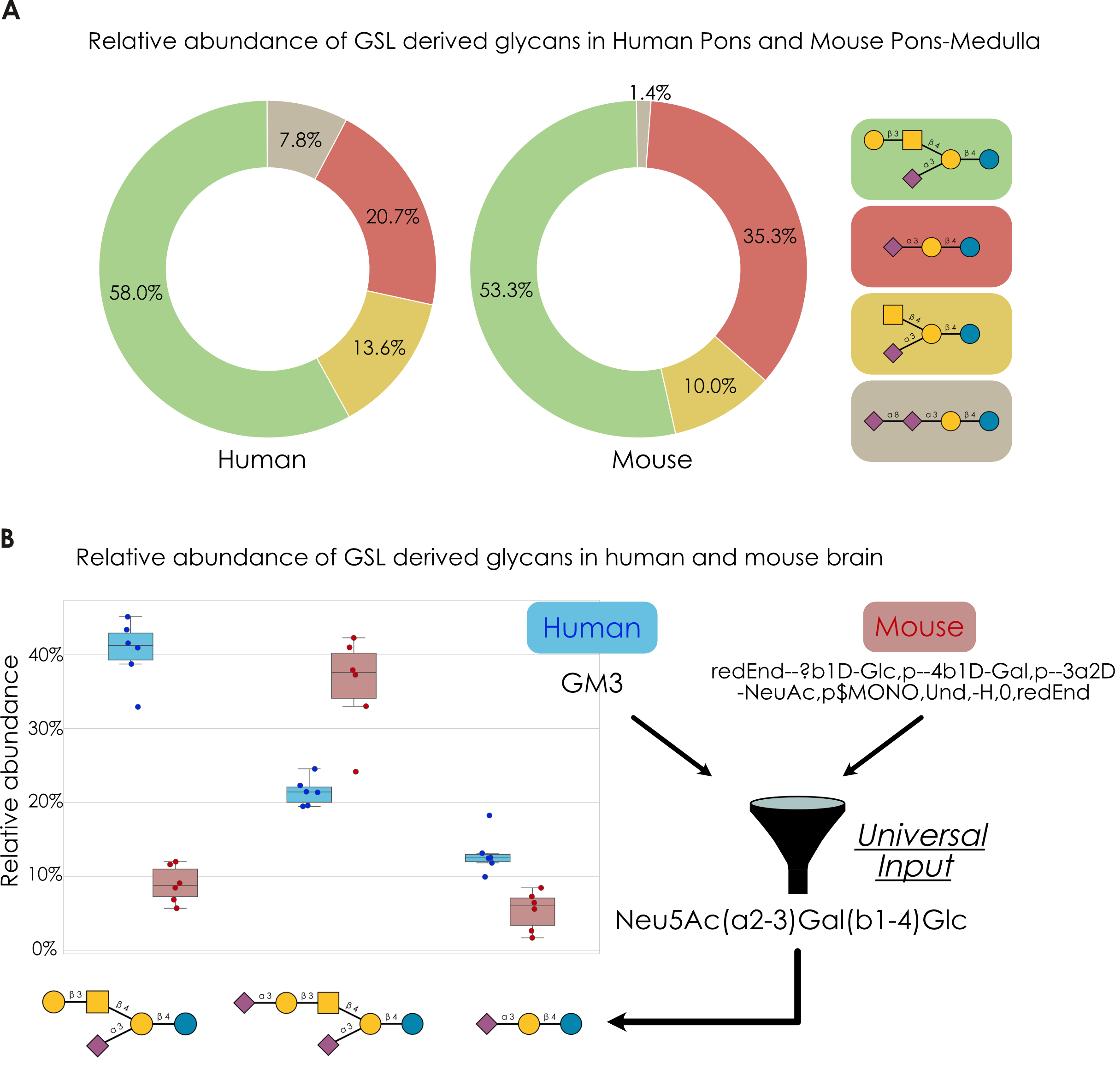
**

**Supplementary Figure 4. Universal input enables analysis of results recorded in different languages. A)** The four shared GSL derived glycans identified in mouse pons-medulla (Lee et al 2024) and in human pons (Schindler et al 2025) show similar relative abundances. **B)** The three shared GSL derived glycans identified in whole mouse brain lysate (Moh et al 2024) and in human dorsolateral prefrontal cortex (Huang et al 2022) differed in their relative abundances. Due to differences in publication nomenclatures of these datasets, such a comparison would have otherwise required manual transcription of every structure.


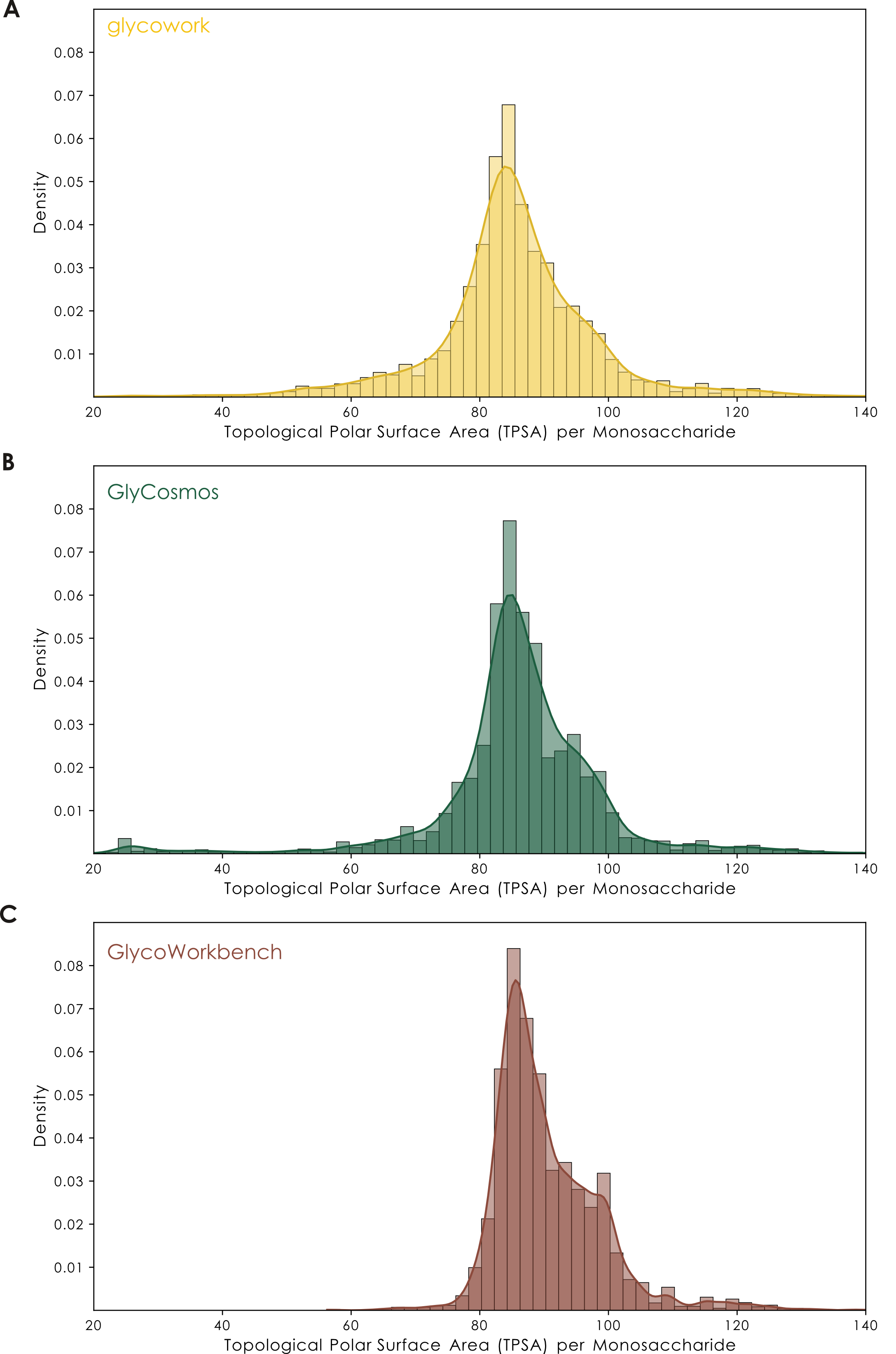
**Supplementary Figure 5. Universal Input enables calculating arbitrary chemical features from fully defined glycans. A-C)** We used 24,626 chemically fully defined glycans from *glycowork.glycan_data.loader.df_glycan* (v1.7.0; IUPAC-condensed), 34,639 chemically fully defined glycans from GlyCosmos (WURCS), and 23,135 chemically fully defined glycans from GlycoWorkbench. Glycan representations from these resources were converted into SMILES string via *canonicalize_iupac* and GlyLES. Then, using rdkit (version 2025.03.2), we calculated the Topological Polar Surface Area (TPSA) of each glycan, and divided the TPSA value by the number of monosaccharides in the glycan.
